# A study of dose-response relationship between tobacco habits and oral leukoplakia.

**DOI:** 10.1038/bjc.1984.210

**Published:** 1984-10

**Authors:** P. C. Gupta

## Abstract

In a house-to-house survey in Ernakulam district, Kerala, India, 12,213 tobacco users were interviewed about the details of their tobacco usage and examined for the presence of leukoplakia. The frequency of tobacco habit was associated with the prevalence of leukoplakia indicating a positive dose-response relationship. The dose-response relationship remained significant, taking age, sex, and the type of tobacco habit into account. After adjusting for all these variables jointly the association still remained significant. The dose-response relationship was stronger for the smoking habit than for the chewing habit. A weaker relationship in the chewing habit was not due to the duration of chewing habit or the habit of retaining the betel quid in the mouth while sleeping. Thus the dose-response relationship, although significant, was different for tobacco smoking and chewing habits.


*Br. J. Cancer* (1984), **50**, 527–531

### A study of dose-response relationship between tobacco habits and oral leukoplakia

P.C. Gupta

*Basic Dental Research Unit, Tata Institute of Fundamental Research, Homi Bhabha Road, Bombay 400 005, India.*

**Summary** In a house-to-house survey in Ernakulam district, Kerala, India, 12,213 tobacco users were interviewed about the details of their tobacco usage and examined for the presence of leukoplakia. The frequency of tobacco habit was associated with the prevalence of leukoplakia indicating a positive dose-response relationship. The dose-response relationship remained significant, taking age, sex, and the type of tobacco habit into account. After adjusting for all these variables jointly the association still remained significant. The dose-response relationship was stronger for the smoking habit than for the chewing habit. A weaker relationship in the chewing habit was not due to the duration of chewing habit or the habit of retaining the betel quid in the mouth while sleeping. Thus the dose-response relationship, although significant, was different for tobacco smoking and chewing habits.

The association between oral leukoplakia and tobacco habits is well established in numerous epidemiologic studies. The association has generally been found to be strong and therefore the habits of tobacco smoking and chewing are accepted as the principal aetiologic factors for oral leukoplakia (Pindborg, 1980).

If the association is examined as a causal one according to the established principles in epidemiology the hypothesis is confirmed on almost all counts for which the data are available; for example, the association is biologically plausible, has been found to be quite consistent in different population groups (Pindborg *et al.*, 1968; Mehta *et al.*, 1969, 1972; Banoczy, 1980), as well as in different studies in similar population groups (Pindborg *et al.*, 1967; Mehta *et al.*, 1969; Roed Petersen *et al.*, 1972), is confirmed through prospective studies (Mehta *et al.*, 1972; Gupta *et al.*, 1980) and when tobacco habits are discontinued a significant increase in the regression of leukoplakia is observed (Mehta *et al.*, 1982).

An important criterion for examining the causal hypothesis is the relationship between the disease and the degree of exposure to the risk factor or the dose-response relationship. Some studies have reported the dose-response relationship between oral leukoplakia and tobacco habits, (Dayal *et al.*, 1978; Baric *et al.*, 1982) however, they have not controlled for confounding variables in their analysis. In this paper, the dose-response relationship between oral leukoplakia and tobacco habits is investigated controlling the effects of confounding variables.

Received 18 April 1984; accepted 25 June 1984

### Subjects and methods

The district of Ernakulam in Kerala State was chosen for this study as the habits of chewing betel quid and smoking bidis were known to be widespread in this district. In a house-to-house survey 12,213 tobacco users aged 15 years and above were interviewed about their tobacco habits and examined for the presence of oral leukoplakia. Leukoplakia was defined as a raised white or greyish white patch 5 mm or more in diameter which could not be rubbed off and could not be attributed to any other diagnosable disease. This definition did not carry any histological connotation. The methodology of the survey was the same as that given by Mehta *et al.* (1969).

In the study sample bidi smoking and pan chewing were the most common forms of tobacco usage. Bidi is a cheap smoking stick made by rolling a dried piece of temburni leaf (Diospyrous melanoxylon) into a conical shape and securing the roll with thread. The length of a bidi varies from 4 to 8 cm and it contains 0.15 to 0.25 g of coarse powdered tobacco. Pan is a quid consisting of betel leaf, arecanut, lime (calcium hydroxide) and tobacco. The usage of cigarette and tobacco alone or tobacco with lime was infrequent (<7%) and therefore in this paper these habits have not been categorized separately.

The tobacco users were asked about the duration and the frequency of their tobacco habit. The chewers were also questioned about the habit of retaining the betel quid in the mouth while retiring for sleep. For these who smoked as well as chewed details about both habits were recorded. The duration of tobacco habit was defined as the number of years for which the individual had been

© The Macmillan Press Ltd., 1984

using tobacco for smoking or chewing. The frequency of tobacco habit was defined as the number of bidis smoked per day for smokers and the number of betel toabcco quids chewed per day for chewers. For simplicity of presentation, at times frequency data for smokers and chewers were combined. This, however, did not imply any assumption of equivalence between the dose represented by bidi and betel tobacco quid.

Relative risks of dose-response were calculated by dividing the prevalence of leukoplakia in the higher frequency group by the prevalence of leukoplakia in the lower frequency group.

### Results

Among 12,213 individuals examined, 10,490 practised a single habit of either chewing or smoking and the rest of 1,723 practised chewing as well as smoking habits. Table I shows the distribution of the frequency of tobacco habit for 10,490 individuals who practised a single smoking or chewing habit and the prevalence of leukoplakia per 1,000. There is a clear and significant increase in prevalence with increase in the frequency of tabacco habit. To simplify further analysis and to avoid the problem of small numbers only two frequency classes: 1–10 and 11 and above, are given in the subsequent tables.

**Table I** Prevalence of leukoplakia according to frequency of tobacco chewing or smoking habit per day.

| *Bidis smoked or quids chewed per day* | *No. of persons* | *Persons with leukoplakia* | *Prevalence per 1000* |
|---|---|---|---|
| 1–5 | 2212 | 25 | 11.3 |
| 6–10 | 4001 | 56 | 14.0 |
| 11–15 | 1519 | 43 | 28.3 |
| 16 and above | 2758 | 93 | 33.7 |
| Total | 10490 | 217 | 20.7 |

$P<0.01$.
$\chi^2=45.9$, df=3.

Table II shows the age distribution of frequency of tobacco habits, prevalence of leukoplakia, and relative risks. Lower frequency was more common in older individuals and higher frequency was more common among younger individuals. For each age group, however, the prevalence of leukoplakia was significantly higher in the frequency group 11 and above compared to the frequency group 1 to 10 showing that the relative risks were significant.

Table III shows the distribution of the frequency of tobacco habit, the prevalence of leukoplakia and the relative risk according to sex and according to the type of tobacco habits. In each category the prevalence of leukoplakia was higher in the frequency group 11 and above compared to the frequency group 1 to 10. Except for females, the relative risk was highly significant for all other categories.

Table II demonstrates that frequency of tobacco habit was associated with age ($\chi^2=1124$, df=5, $P<0.001$) and Table III with sex ($\chi^2=2375$, $P<0.001$) and with the type of tobacco habit (smoking, chewing) ($\chi^2=3842$, $P<0.001$). It is known that the occurrence of leukoplakia is also strongly associated with these three variables (Pindborg, 1980) and among these three variables age and the type of tobacco habit and sex and the

**Table II** Prevalence of leukoplakia according to frequency of tobacco habit (chewing or smoking) and age.

| | *Bidis smoked or quids chewed per day* | | | | |
|---|---|---|---|---|---|
| | *1–10* | | *11 and above* | | |
| *Age group* | *No. of persons* | *Prevalence per 1000* | *No. of persons* | *Prevalence per 1000* | *Relative risk* |
| 15–24 | 786 | 0 | 730 | 4.1 | — |
| 25–34 | 764 | 2.6 | 1222 | 10.6 | 4.1* |
| 35–44 | 1076 | 10.2 | 1115 | 37.7 | 3.7** |
| 45–54 | 1266 | 22.9 | 690 | 73.9 | 3.2** |
| 55–64 | 1106 | 19.9 | 327 | 52.0 | 2.6** |
| 65 and above | 1215 | 14.0 | 193 | 51.8 | 3.7** |
| Total | 6213 | 13.0 | 4277 | 31.8 | 2.4** |

**$P<0.01$.
*$P<0.05$.

Table III Prevalence of leukoplakia according to frequency, sex, and type of tobacco habit.

| | *Bidis smoked or quids chewed per day* | | | | |
|---|---|---|---|---|---|
| | *1–10* | | *11 and above* | | |
| | *No. of persons* | *Prevalence per 1000* | *No. of persons* | *Prevalence per 1000* | *Relative risk* |
| Sex | | | | | |
| Males | 2974 | 14.8 | 4002 | 32.7 | 2.2** |
| Females | 3239 | 11.4 | 275 | 18.2 | 1.6 |
| Tobacco habit | | | | | |
| Smoking | 2055 | 5.8 | 3821 | 31.1 | 5.4** |
| Chewing | 4158 | 16.6 | 456 | 37.3 | 2.2** |

**$P<0.01$.

**Table IV** Age-adjusted prevalence of leukoplakia according to frequency, sex and type of tobacco habit.

| | *Bidis smoked or quids chewed per day* | | | | | | |
|---|---|---|---|---|---|---|---|
| | *1–10* | | | *11 and above* | | | |
| *Sex* | *No. of persons* | *Persons with leukoplakia* | *Age-adjusted prevalence per 1000* | *No. of persons* | *Persons with leukoplakia* | *Age-adjusted prevalence per 1000* | *Relative risk* |
| Smoking | | | | | | | |
| Males | 1915 | 10 | 8.9 | 3807 | 119 | 44.9 | 5.0** |
| Females | 140 | 2 | 9.9 | 14 | — | — | — |
| Chewing | | | | | | | |
| Males | 1059 | 34 | 26.6 | 195 | 12 | 49.1 | 1.8* |
| Females | 3099 | 35 | 8.4 | 261 | 5 | 14.6 | 1.7 |
| Males & females | 4158 | 69 | 12.1 | 456 | 17 | 23.2 | 1.9* |

**$P<0.01$.
*$P<0.05$.

type of tobacco habit are associated with each other (Mehta *et al.,* 1969). To eliminate possible confounding effects of there relationships on the association of leukoplakia and frequency of tobacco habit, Table IV shows the age adjusted prevalence of leukoplakia and the relative risk according to sex and type of tobacco habit. For males who smoked the relative risk (5.0) was highly significant and those those who chewed the relative risk (1.8) was just significant. For females who smoked, sufficient observations were not available and for those who chewed the relative risk (1.7) was not significant. Thus the dose-response relationship appeared to be stronger for smoking habit than for chewing habit.

To probe this phenomenon further, prevalence of leukoplakia was analysed by another component of dose for chewers, the habit of retaining the betel quid in the mouth while sleeping (Table V). It is clear that for females as well as for males there was no significant difference in prevalences.

**Table V** Prevalence of leukoplakia according to sex and the habit of retaining the quid in the mouth while sleeping.

| *Sex* | *Retained quid while sleeping* | *No. of chewers* | *Persons with leukoplakia* | *Prevalence per 1000* |
|---|---|---|---|---|
| Males | Yes | 96 | 3 | 31.3 |
| | No | 1158 | 43 | 37.1 |
| Females | Yes | 383 | 4 | 10.4 |
| | No | 2977 | 36 | 12.1 |

Table VI looks into the possibility of the differences being confounded by the duration of chewing habit. The prevalences did not differ significantly in different duration groups. Age-adjusted prevalences (not shown in Table VI) did not reveal any pattern either. In the study sample most individuals tended to start their tobacco habits at similar age resulting in a high correlation between age and the duration of the habit (correlation coefficient 0.6). Adjusting for age, therefore, also adjusted for the duration of the tobacco habit to a considerable extent.

**Table VI** Prevalence of leukoplakia according to the duration of tobacco chewing habit and sex.

| *Duration of tobacco chewing habit* | *Males* | | *Females* | |
|---|---|---|---|---|
| | *No. of tobacco chewers* | *Prevalence per 1000* | *No. of tobacco chewers* | *Prevalence per 1000* |
| 10 years or less | 195 | 41.0 | 1098 | 10.9 |
| 11–30 years | 293 | 44.4 | 1104 | 12.7 |
| 31 years or more | 642 | 32.7 | 873 | 11.5 |

$P<0.05$

$\chi^2=0.9$ for males and 0.3 for females.

Another possible reason for a higher relative risk of dose response among smokers could be a more accurate assessment of frequency by smokers compared to chewers. The relative risks of dose response was therefore computed for 1,723

individuals who smoked as well as chewed. Among smokers of 1 to 10 bidis per day the relative risk for chewing over 10 quids was 1.1 ($P<0.05$) and among chewers of 1–10 quids the relative risk for smoking of over 10 bidis was 2.0 ($P<0.05$).

### Discussion

In general "dose" or a measure of the degree of exposure to a risk factor can consist of several components. In the context of the present study, apart from the frequency and the duration of tobacco habit, several other components, specific to the type of tobacco habit, can be considered as representing, or at least affecting the dose. For example, for chewing habit, the duration for which a quid is kept in the mouth, amount of tobacco in a quid, the type of tobacco used, and for smoking habits, the degree of inhalation, the frequency of puffs, the left-over length of the butt, etc. could be considered as important components of dose in different circumstances. In the present study although information was collected on some of these components either there was not enough variation to justify separate analysis (type of tobacco by local names) or the information was not considered reliable enough (duration of keeping the quid in the mouth). Information on other aspects could not be collected. It is unlikely that the duration of keeping the quid would show any significant difference for the risk of leukoplakia because the habit of retaining the quid in the mouth while sleeping which effectively categorises the duration of keeping quid to less than 8 hours and more than 8 hours, did not show any difference.

The study shows that the dose-response relationship between leukoplakia and tobacco habits is significant after adjusting for age, sex and the type of tobacco habit. The striking result from this study, however, is that the dose-response relationship is stronger for smokers compared to chewers, and the difference is not attributable to the duration of chewing habit, habit of retaining quid in the mouth while sleeping, or better recall of frequency by smokers compared to chewers. The difference in the strength of the dose-response relationship could not be attributed to the choice of the cut-off point either.

It is interesting that another study from India which reported on the association between prevalence of leukoplakia and the frequency and the duration of chewing habit (Dayal *et al.*, 1978) showed an increasing trend in the prevalence with increase in the duration and increase in the frequency. No statistical tests of significance were reported.

The stronger dose-response relationship for smokers than for chewers although, inexplicable, may not be surprising. It has been reported before that leukoplakia associated with smoking habit and leukoplakia associated with chewing behave differently with regard to incidence, spontaneous regression and malignant transformation (Mehta *et al.*, 1981). It is therefore feasible that the two types of tobacco habit should show different results for dose-response relationships.

I am grateful to Dr Fali S. Mehta and Prof. J.J. Pindborg for their valuable assistance and to Drs R.B. Bhonsle and P.R. Murti for the field work.

The research conducted for this paper was supported in whole by funds from the National Institutes of Health, USA under a P.L. 480 grant, research agreement no. 01-022-N.

### References

BANOCZY, J. (1982). *Oral Leukoplakia,* Budapest, Akademiai Kiado.

BARIC, J.M., ALMAN, J.E., FELDMAN, R.S. & CHAUNCEY, H.H. (1982). Influence of cigarette, pipe and cigar smoking, removable partial dentures and age on oral leukoplakia. *Oral Surg.,* **54,** 424.

DAYAL, P.K., MANI, N.J. & BHARGAVA, K. (1978). Prevalence of oral cancer and precancerous lesions in "Pan/Supari" chwers. *Indian J. Public Health,* **22,** 234.

GUPTA, P.C., MEHTA, F.S., DAFTARY, D.K. & 16 others. (1982). Incidence rates of oral cancer and natural history of oral precancerous lesions in a 10 year follow-up study of Indian villagers. *Commun. Dent. Oral Epidemiol.,* **8,** 287.

MEHTA, F.S., AGHI, M. B., GUPTA, P.C. & 4 others. (1982). An intervention study of oral cancer and precancer in rural Indian populations. A preliminary report. *Bull. W.H.O.,* **60,** 441.

MEHTA, F.S., GUPTA, P.C., DAFTARY, D.K., PINDBORG, J.J. & CHOKSI, S.K. (1972). An Epidemiologic study of oral cancer and precancerous conditions among 101,761 villagers in Maharashtra, India. *Int. J. Cancer,* **10,** 134.

MEHTA, F.S., GUPTA, P.C. & PINDBORG, J.J. (1981). Chewing and smoking habits in relation to precancer and oral cancer. *J. Cancer Res. Clin. Oncol.,* **99,** 44.

MEHTA, F.S., PINDBORG, J.J., GUPTA, P.C. & DAFTARY, D.K. (1969). Epidemiologic and histologic study of oral cancer and leukoplakia among 50,915 villagers in India. *Cancer,* **24,** 832.

MEHTA, F.S., SHROFF, B.C., GUPTA, P.C. & DAFTARY, D.K. (1972). Oral Leukoplakia in relation to tobacco habits. A ten-year follow-up study of Bombay policemen. *Oral. Surg.,* **34,** 426.

PINDBORG, J.J. (1980). *Oral Cancer and Precancer,* Bristol: John Wright & Sons Ltd.

PINDBORG, J.J., BARMES, O.D. & ROED-PETERSEN, B. (1968). Epidemiology and histology of oral leukoplakia and leukodema among Papuans and New Guineans. *Cancer,* **22,** 379.

PINDBORG, J.J., KIAER, J., GUPTA, P.C. & CHAWLA, T.N. (1967). Studies in oral leukoplakia. Prevalence of leukoplakia among 10,000 persons in Lucknow, India with special reference to use of tobacco and betel nut. *Bull. W.H.O.,* **37,** 109.

ROED-PETERSEN, B., GUPTA, P.C., PINDBORG, J.J., & SINGH, B. (1972). Association between oral leukoplakia and sex, age and tobacco habits. *Bull. W.H.O.,* **47,** 13.


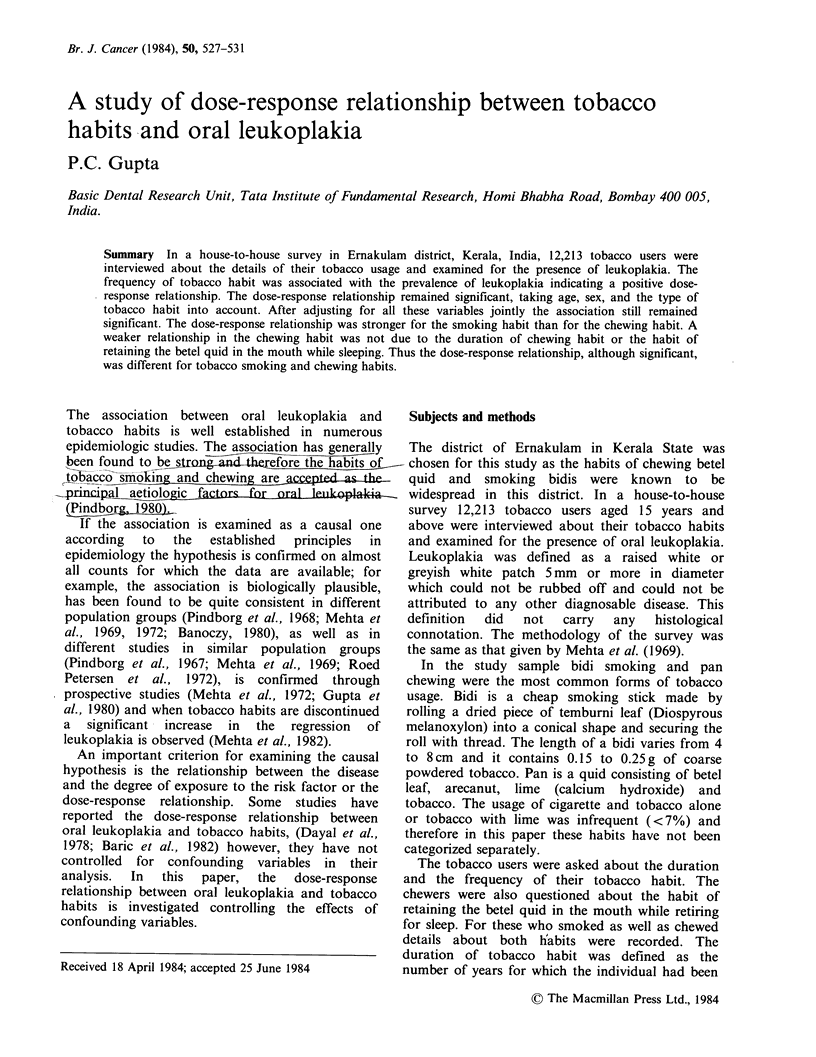

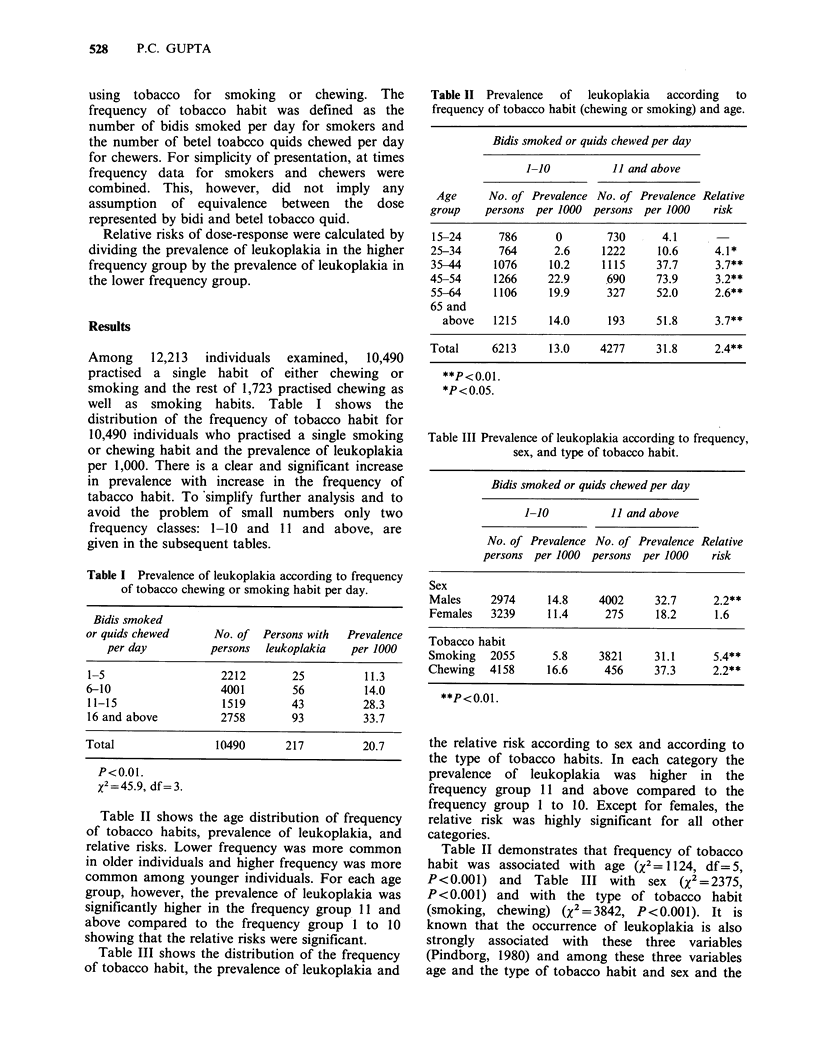

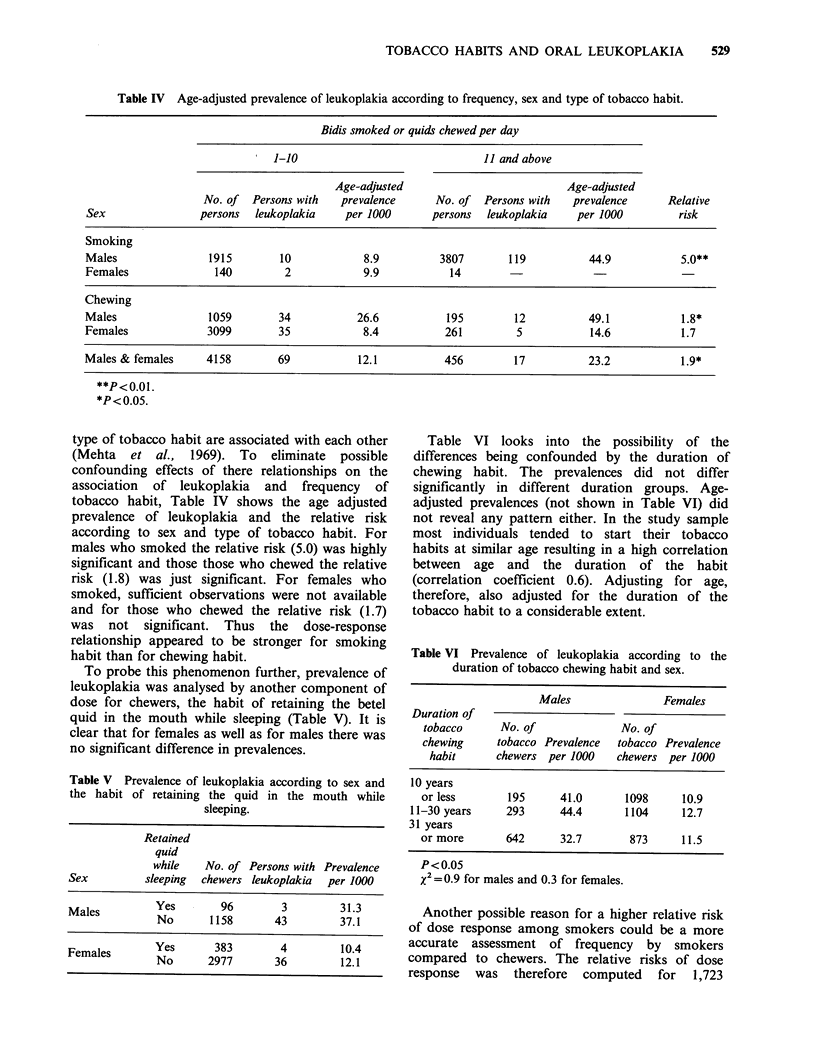

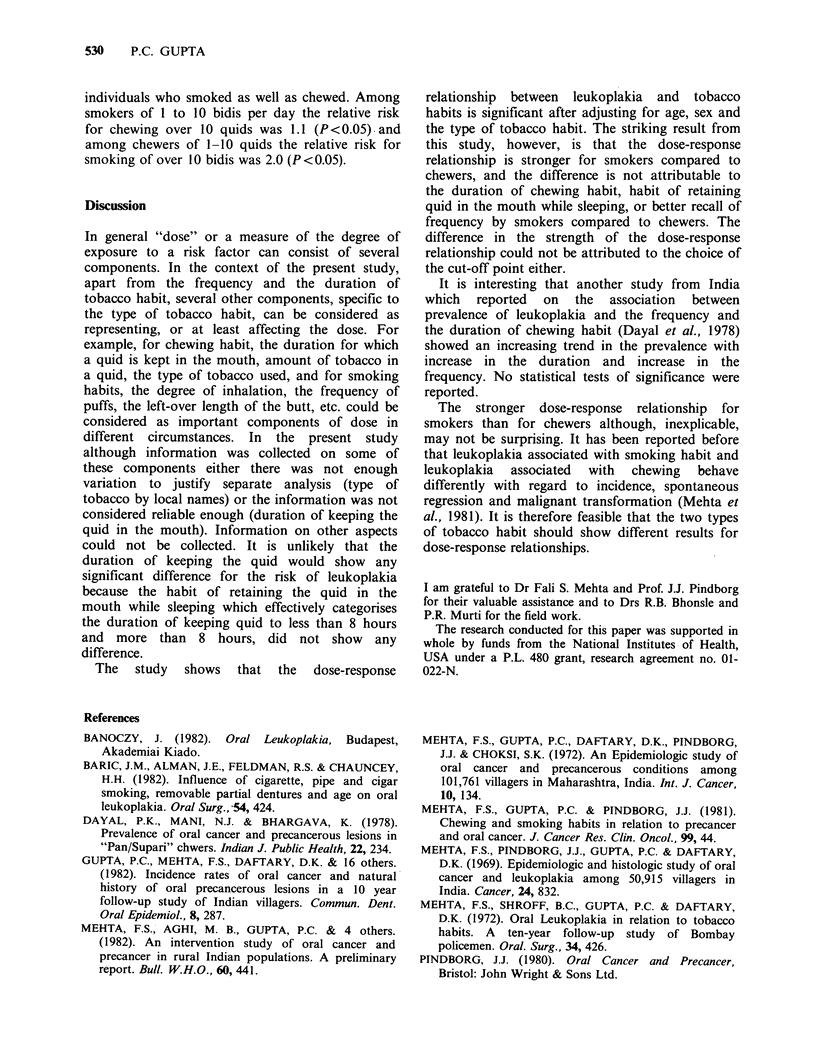

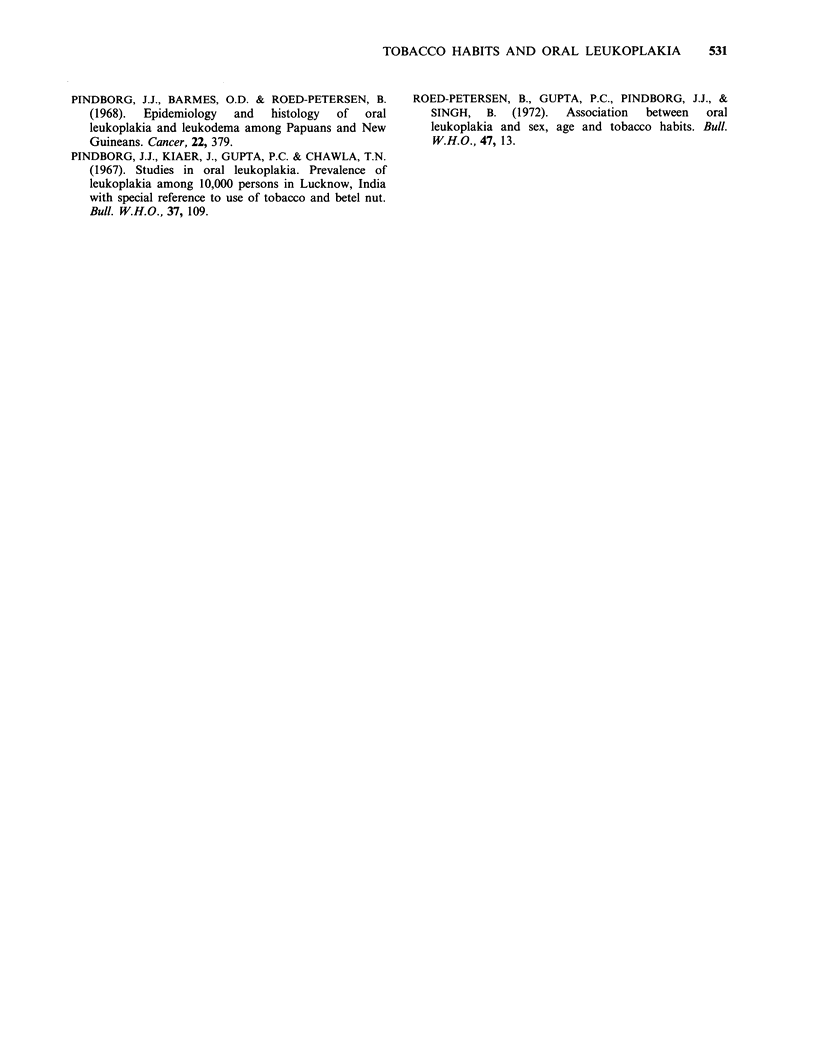


## References

[OCR_00399] Baric J. M., Alman J. E., Feldman R. S., Chauncey H. H. (1982). Influence of cigarette, pipe, and cigar smoking, removable partial dentures, and age on oral leukoplakia.. Oral Surg Oral Med Oral Pathol.

[OCR_00405] Dayal P. K., Mani N. J., Bhargava K. (1978). Prevalence of oral cancer and precancerous lesions in 'pan'/'supari' chewers.. Indian J Public Health.

[OCR_00417] Mehta F. S., Gupta M. B., Pindborg J. J., Bhonsle R. B., Jalnawalla P. N., Sinor P. N. (1982). An intervention study of oral cancer and precancer in rural Indian populations: a preliminary report.. Bull World Health Organ.

[OCR_00423] Mehta F. S., Gupta P. C., Daftary D. K., Pindborg J. J., Choksi S. K. (1972). An epidemiologic study of oral cancer and precancerous conditions among 101,761 villagers in Maharashtra, India.. Int J Cancer.

[OCR_00435] Mehta F. S., Pindborg J. J., Gupta P. C., Daftary D. K. (1969). Epidemiologic and histologic study of oral cancer and leukoplakia among 50,915 villagers in India.. Cancer.

[OCR_00441] Mehta F. S., Shroff B. C., Gupta P. C., Daftary D. K. (1972). Oral leukoplakia in relation to tobacco habits. A ten-year follow-up study of Bombay policemen.. Oral Surg Oral Med Oral Pathol.

[OCR_00453] Pindborg J. J., Barmes D., Roed-Petersen B. (1968). Epidemiology and histology of oral leukoplakia and leukoedema among Papuans and New Guineans.. Cancer.

[OCR_00459] Pindborg J. J., Kiaer J., Gupta P. C., Chawla T. N. (1967). Studies in oral leukoplakias. Prevalence of leukoplakia among 10,000 persons in Lucknow, India, with special reference to use of tobacco and betel nut.. Bull World Health Organ.

[OCR_00466] Roed-Petersen B., Gupta P. C., Pindborg J. J., Singh B. (1972). Association between oral leukoplakia and sex, age, and tobacco habits.. Bull World Health Organ.

